# How does head position affect laryngeal vision with a video laryngeal mask airway?

**DOI:** 10.3389/fmed.2024.1469225

**Published:** 2024-12-16

**Authors:** Caridad G. Castillo-Monzón, Hugo Antonio Marroquín-Valz, Tomasz Gaszynski, Manuel Cayuela, Javier Orozco, Pawel Ratajczyk

**Affiliations:** ^1^Service of Anaesthesiology, Reanimation and Pain Therapy, University General Hospital of Cartagena-Murcia, Cartagena, Spain; ^2^University General Hospital of Cartagena-Murcia, Cartagena, Spain; ^3^Department of Anaesthesiology and Intensive Therapy, Medical University of Lodz, Łódź, Poland

**Keywords:** supraglottic airway device, head position, glottis view, videolaryngeal mask, vision mask

## Abstract

**Background:**

The Laryngeal Mask Airway Vision Mask (LMA VM) is a supraglottic airway device (SAD) with a vision guidance system. The ideal head and neck position for direct laryngoscopy is known, but the ideal position for placing a LMA is not. The objective of this study is to evaluate and compare the optimal position for placement of a video laryngeal mask airway.

**Methods:**

This prospective, observational, transversal, and analytical study was performed in 72 consecutive patients. In the same patient, laryngeal vision was first assessed with the head and neck in the sniffing position and then with the head in the neutral position. Procedures were performed by the same investigator. The assessment of the laryngeal view was performed using two classifications: Cormack–Lehane classification and Brimacombe classification. The placement of the device was considered adequate when the Cormack–Lehane rating was between 1 and 2 and the Brimacombe rating between 2 and 4.

**Results:**

In this study, 72 patients participated. In the assessment of the glottis using the Cormack-Lehane classification for fibre-optic view, laryngeal visibility was adequate in 64 (88.89%) patients in the neutral position and in 65 (90, 28%) patients in the sniffing position (*p* > 0.05). In the fibre-optic view of the glottis, evaluated using the Brimacombe classification, laryngeal visibility was adequate in 68 (93%) patients in the neutral position and in 69 (95%) patients in the sniffing position (*p* > 0.05). There was no statistically significant difference in the rate of success between the sniffing position (70 patients, 97.22% success rate) and the neutral position (67 patients, 93.06% success rate) during the first insertion attempt. Two patients required a second attempt in the sniffing position, while five patients required a second attempt in the neutral position.

**Conclusion:**

An adequate sniffing position did not result in a better glottic view than the neutral position. Additional manoeuvres were equal in both positions. The head–neck position does not influence on the placement of a third-generation SAD.

## 1 Introduction

Supraglottic airway devices (SADs) offer an option for an alternative airway management to traditional tracheal intubation and carry potential beneficial effects such as ease of placement and less airway disturbance (1).

The first SAD introduced to the market were the Laryngeal Mask Airway (LMA) over 40 years ago. Timmermann et al. classified the SADs in 2011 (2) into two generations: first-generation devices with only a breathing channel for ventilation and second-generation devices with two channels: breathing and a gastric channel to evacuate the gastric content and potentially protect against aspiration, also with better sealing pressures. Van Zunderck suggested calling those with channel for built-in video as third-generation SADs (3).

Currently, we know that proper head and neck position is essential to achieve adequate visualisation of the larynx during direct laryngoscopy, a concept that was first described by Kirstein in 1895 (4). The evolution of research has culminated in the current standard practice of direct laryngoscopy in the sniffing position, which aligns the ear lobe and sternal fork. The elevation of the head in the sniffing position may vary between individuals depending on the length of the neck, the anterior posterior diameter of the thorax, and the size and shape of the head in relation to the thorax. Moreover, the height of the pillow that is used depends on the patient’s anatomy (5). There is no standard pillow that works for all patients.

In their study, El-Orbany et al. (6) employed three distinct head positions for direct laryngoscopy: no elevation, a sniffing position with a 6-cm elevation (neck flexion of approximately 35°), and a sniffing position with a 10-cm pillow elevation of the occiput (neck flexion ≥35°). Laryngoscopy was found to difficult when the head was not elevated (8.3%), when the sniffing position was used (2.39%), and when the head elevation was higher (1.19%).

The optimal head and neck position for direct laryngoscopy has been extensively studied and documented. To the best of our knowledge, there are scarce reports on the association of head elevation degree and LMA insertion technique and its efficiency (7). Changes in head and neck position may significantly affect the performance of SAD by altering the pharyngeal structure (8).

A standard method recommended for the insertion of for the standard insertion method of the LMA is the extension of the head and the flexion of the neck (9). Brimacombe and Berry found no significant difference in the success rate of insertion when they compared the sniffing position with the neutral position (10).

Most studies assess whether the ideal anatomical position for SADs is achieved with clinical parameters, laryngoscopy (11, 12), videolaryngoscopy (13) or fibroscopy (14–16). However, all of these alternatives can delay and complicate the procedure because of the additional time required.

Within the clinical parameters, the oropharyngeal leak pressure (OPLP) (17, 18) is commonly measured during LMA insertion to evaluate the degree of airway protection (19). Kim et al. (17) noted that OPLP indicates clinical performance or function of the LMA better than the fibre-optic score system does.

Improper placement of the SAD may result in partial or complete obstruction of the airway. On the other hand, excessive LMA cuff pressure may exceed the capillary tissue perfusion pressure. The manufacturer recommends that the pressure not exceed 60 cm H_2_O (44 mmHg) when using these devices.

As a standard practice, the placement of a SAD is a blind procedure with the belief that its placement leads to a proper positioning. However, the literature suggests that despite suggested clinical tests to verify proper placement, 50–80% of the times, its placement is deficient (14). Blind insertion of a SAD can lead to misplacement and leak during ventilation efforts.

The Cormack-Lehane (CL) classification (1984) is the gold standard for direct laryngoscopy. There is no universally accepted definition for difficult intubation. In most studies, difficult intubation was defined as a CL grade 3 and 4 (20), with its occurrence varying between 0.3 and 13% (21).

In 1993, Brimacombe and Berry introduced a fibre-optic scoring system to standardise the assessment of SAD position, which is being most widely used since then (22). Fibre-optic scores of 2, 3, and 4 are anatomically acceptable placements and 1 is considered a poor placement (23). The score 1 of was found to occur 9.4% of the times (24, 25).

Vision Mask is a video laryngeal mask system, with continuous vision and five accesses developed by Pedro Acha MD, the inventor of Airtraq and Totaltrack. This mask was presented for the first time in the world at the Anaesthesia Service of the University Hospital Complex of Cartagena, Murcia, in Spain and we have been using it since November 2021. It is manufactured by Integral Medical Products Co., Ltd., Yuecheng District, Shaoxing, China. It is distributed in Spain by Productos Medicos Hospitalarios (PMH), Molina de Segura (Murcia).

These devices combine the advantages of an integrated video laryngoscope incorporated into a second-generation SAD. This device comes with the additional benefit of vision, which is not available on all laryngeal masks currently available on the market. The SAD with a vision guidance system during its placement allows for the evaluation of the larynx, the objectification of the spasm of the vocal cords, bronchial aspiration, in addition to serving as a visual aid for intubation and allowing standard ventilation (26).

Airway management is an area of ongoing research, and video laryngeal mask system is a technology that is here to stay like the ultrasound scan.

In this study, we aimed to evaluate the hypothesis that sniffing position with axis alignment allows for a better placement and positioning of the Vision Mask device and better vision of the glottic area when compared to the neutral position. The secondary objectives were to compare the number of placement attempts, additional manoeuvres in device placement, haemodynamic response, and complications arising during the procedure.

## 2 Materials and methods

**Figure 1:**
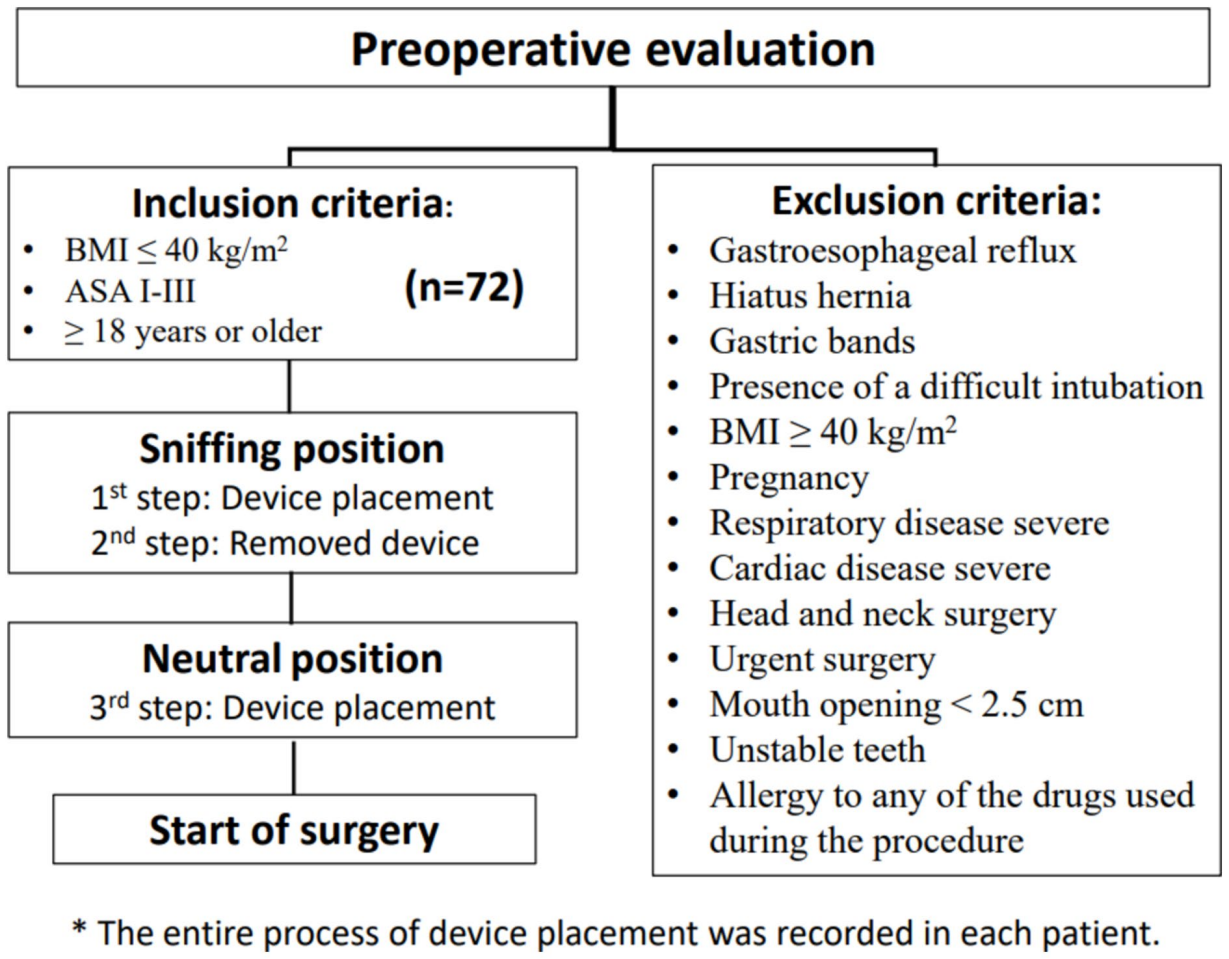
Flow chart of the study.

The study was conducted according to the guidelines of the Declaration of Helsinki and approved by the Institutional Ethics Committee of the Santa Lucia Hospital, Murcia, Spain (CEI. 22-38) on May 31, 2022. Informed written consent was obtained from each patient. A prospective, observational, transversal and analytical study was performed (Figure 1). We prospectively included 72 consecutive patients. The inclusion criteria were as follows: body mass index (BMI) ≤ 40 kg/m (2), American Society of Anaesthesiologists I–III (ASA) physical status, 18 years or older undergoing a variety of surgical interventions were enrolled in the study, and attended by the main investigator, with more than 30 uses of the device at the beginning of the investigation.

The exclusion criteria were the following: patients who had gastroesophageal symptomatic reflux, hiatus hernia, gastric bands, presence of a difficult intubation, BMI greater than 40 kg/m^2^, pregnancy, severe respiratory or cardiac disease, patients undergoing head and neck surgery, urgent surgery, mouth opening less than 2.5 cm, unstable teeth, and allergy to any of the drugs used during the procedure.

Preoperative evaluation of the patient included: age, height, weight, ASA physical status class, Mallampatti classification scored 1 to 4 (obtained with the patient in the sitting position, tongue out without phonation); thyromental distance (measured with the patient in the sitting position) being classified as easy when ≥6 cm and difficult when <6 cm, with head in extension; interincisor distance, easy when ≥4 cm and difficult when <4 cm, and neck circumference. Neck circumference was measured with the head in a neutral position, at the level of the thyroid cartilage, and it was considered easy when it was <45 cm and difficult when ≥45 cm.

**Figure 2:**
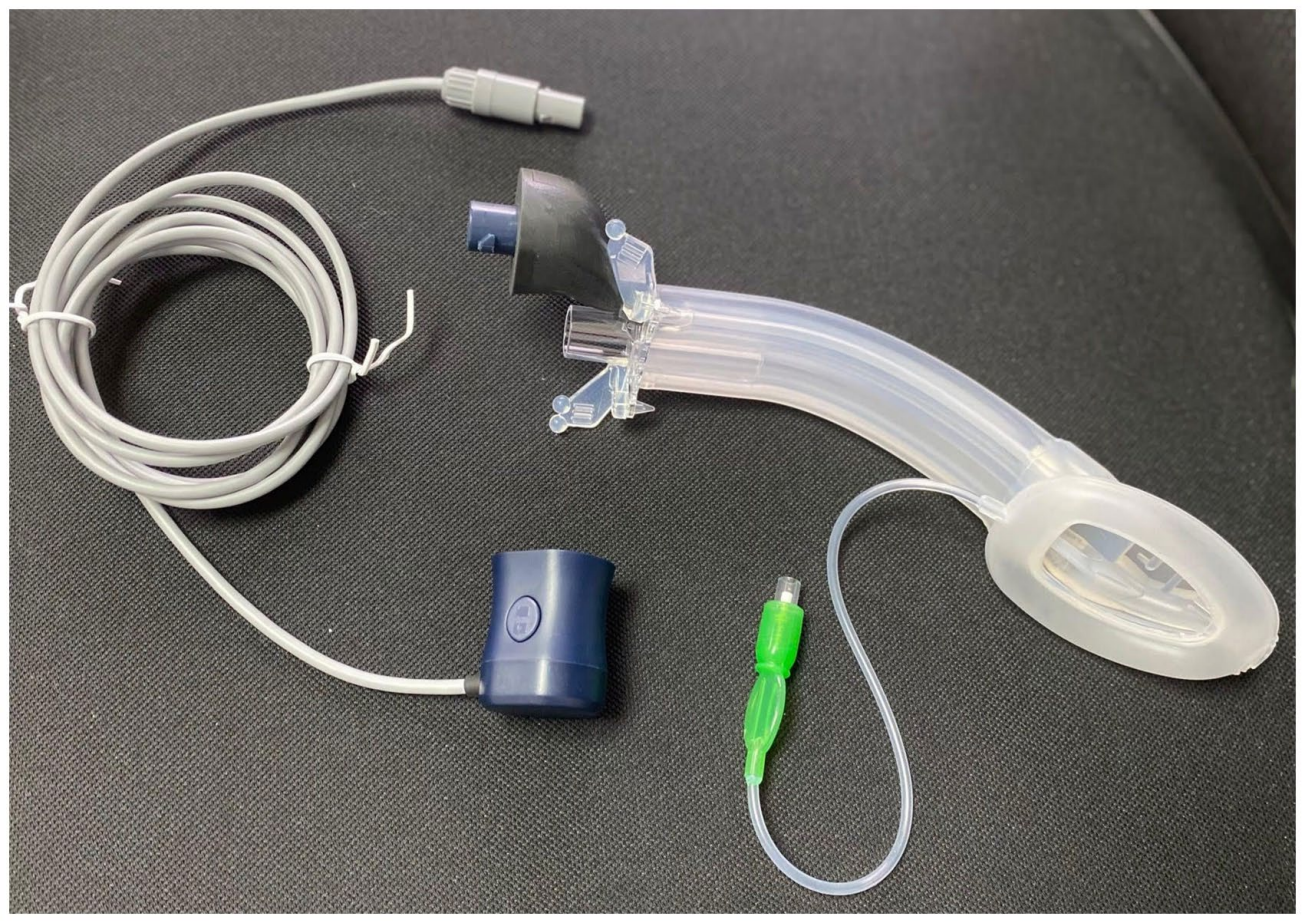
Vision mask size 3 with connection.

Vision mask consists of five access points (26):

1. An access point which allows measurement with a manometer to determine the pressure inside the laryngeal mask in cmH_2_O, where the pressure ranges from 10 to 20 mmHg.
2. A video stylus channel with connection. A left side access point is available for its camera stylet, which can be connected to a reusable 2.8-inch and 7-inch portable monitor with image/video recording capabilities. The channel is open at one end and closed at the other end, and it never comes in contact with the patient.
3. Its central access allows gas inlet and outlet, plus the introduction of an endotracheal tube (ETT) for rescue intubation or fibro or a video bronchoscope, with 15 mm connection.
4. The lower right-side access has gastric access. It has an open channel at both ends. Suction catheter number 12 French or lower can be used in Vision Mask size 3 (Figure 2) and suction catheter number 14 French or lower can be used in Vision Mask size 4.
5. An upper right-side access point for a free gas outlet from the interior when using continuous positive airway pressure (CPAP) ventilation.

**Figure 3:**
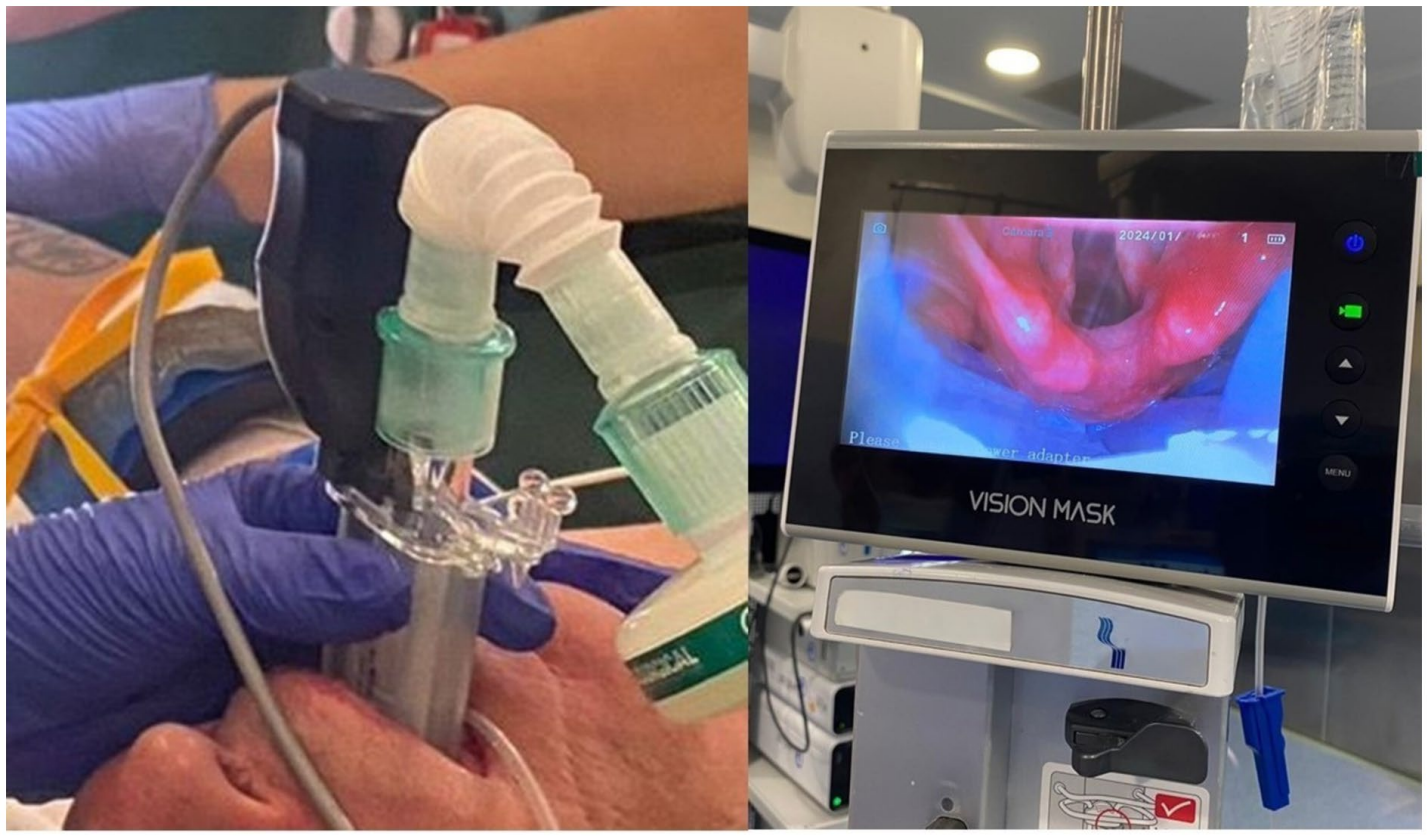
Laryngeal mask airway vision mask: visualisation of entrance to the larynx.

In the same patient, Vision Mask device was placed in the sniffing position (the external auditory meatus and the sternal notch were horizontally aligned with blankets). After the device was removed, the patient’s head and neck were repositioned to the neutral position (head on the operating table). Procedures were performed by the same investigator to avoid bias based on the experience of different operators. All procedures were recorded so that another researcher could evaluate the glottis view in another moment, without him or her having more information about the patient (see Figure 3).

The assessment of the laryngeal view was performed using two classifications: Cormack–Lehane classification and the Brimacombe classification.

The Cormack–Lehane classification is done as follows:

Grade 1: Most of the glottis is visible.

Grade 2: At best, almost half of the glottis is seen at worst only the posterior tip of the arytenoids is seen.

Grade 3: Only the epiglottis is visible.

Grade 4: No laryngeal structures are visible.

The Brimacombe score is decided as follows:

4: Only vocal cords are visible. This is the optimal position.

3: Vocal cords plus the posterior epiglottis are seen.

2: Vocal cords plus the anterior epiglottis are seen.

1: No vocal cords are visible, but function is adequate.

0: Device failure occurs.

Device placement was considered adequate when the Cormack–Lehane rating between 1 and 2 and the Brimacombe rating was between 2 and 4.

The secondary outcome variables were number of attempts, manoeuvres necessary during the insertion to get a better view of the glottic area (external laryngeal manipulation, jaw thrust), haemodynamic response, and complications (incidence of airway trauma: lip or oral mucosa trauma, bleeding into the device at the time of removal, dental trauma, and others).

Three attempts were allowed before a failure of insertion was recorded. If the SAD could not achieve a satisfactory airway within three insertion attempts, an endotracheal tube was inserted for airway management.

### 2.1 Study procedure

After the patients were admitted to premedication area, a peripheral 18G venous access was placed in upper limb and 4–5 mL/kg Ringer’s lactate solution was infused (ideal weight). As part of the procedure, all patients were premedicated with omeprazole 40 mg, dexamethasone 0.1 mg/kg, paracetamol 1 g, and midazolam 1 or 2 mg depending on their needs.

The standard monitoring devices were attached before induction of anaesthesia, including the following: II-lead electrocardiography, non-invasive blood pressure, pulse oximetry, end-tidal carbon dioxide, and bispectral index. Prior to pre-oxygenation, patients were placed first in the sniffing position. Patients were preoxygenated with 100% oxygen with a tight face mask. Induction was initiated when the end tidal of oxygen was more than 90%.

Anaesthesia was induced with 1 mg/kg lidocaine, 2 mg/kg propofol, fentanyl 1 μg/kg, and rocuronium 0.6 mg/kg ideal weight and after target-controlled infusion (TCI) of propofol on the Marsh model: 2 μg/mL during the study using Perfusor® Space TCI Syringe Pump. Atropine 0.5 mg was optional as required.

The choice of mask size and insertion technique was made in accordance with the recommendations of the manufacturer. The posterior surface of the SAD was well lubricated with a water-soluble lubricant. It was left in place and used for the surgical procedure or only for evaluation purposes before the patient was intubated.

Two minutes after the neuromuscular blocking agent was administered, the SAD Vision Mask was placed in the sniffing position. The laryngeal view was filmed and haemodynamic parameters were recorded 1 min before and 1 min after the device was placed. After the Vision Mask device was removed, the patient’s head and neck were repositioned to the neutral position and the SAD was placed. The procedure was recorded.

Adequate ventilator status of the device *in situ* were confirmed by bilateral chest movement, auscultation, and by a normal square wave on the capnogram.

A specific chart was recorded for each patient during the entire procedure.

### 2.2 Statistical analysis

The distributions by fibre-optic scoring of each insertion technique group were compared using the chi-squared test and Fisher exact test when the expected values in any of the cells of a contingency table were below 5 in chi-squared test. In the same way, secondary outcomes variables were measured. A *p*-value <0.05 was considered statistically significant.

As there were no previous studies perfomed with this type of device, to our knowledge, a pilot study was carried out with 25 patients (50 glottic views), obtaining an inadequate vision of 12% in the neutral position and 0% of inadequate vision in the sniffing position, using the Brimacombe classification. With this information, statistical factors that determined the sample size were: proportion of sniffing position (p1) = 0%, proportion of neutral position (p2) = 12%, error alpha = 5%, beta error = 20%, statistical power = 80%, and losses in the study = 15. The calculated sample size was 71 patients.

## 3 Results

A total of 72 patients consented to participate in the study. The patient characteristics are shown in Table 1.

**Table 1:** Patient characteristics (*n* = 72).

Men/Women (*n*, %)	19	26.39%	53	73.61%
Age (years)	53.74	(23–82)	D.S.	13.21
Height (cm)	165.33	(146–192)	D.S.	9.56
Weight (kg)	75.66	(42–129)	D.S.	17.18
ASA physical status				
I	15	20.83%		
II	44	61.11%		
III	13	18.06%		
Body mass index (kg/m^2^)	27.55	(16.00–37.42)	D.S.	5.10
BMI < 30	44	61.11%		
BMI 30 to <35	21	29.17%		
BMI ≥ 35 to <40	7	9.72%		
Mallampati classification				
I	6	8%		
II	37	51%		
III	27	38%		
IV	2	3%		
Tryromental distance (cm)				
≥ 6 cm	59	82%		
< 6 cm	13	18%		
Intercisor gap (cm)				
≥ 4 cm	66	92%		
< 4 cm	6	8%		
Neck circumference (cm)				
≤ 40 cm	54	75%		
41–59 cm	18	25%		
≥ 60 cm	0	0%		
Size vision mask				
3	54	75%		
4	18	25%		
SAD used				
To evaluation	43	60%		
To anaesthesia	29	40%		
Type of surgery				
General Surgery	29	40%		
Gynaecology	19	26%		
Orthopaedic	2	3%		
Urology	12	17%		
Other	10	14%		

The median age of the participants was 53.74 years (range 23–82 years), height 165.33 cm (range 146–192 cm), and weight 75.66 kg (range 42–129 kg) respectively. A majority of the participants were women (53) while the rest of the participants were men (19).

In the assessment of the glottis using the CL classification for fibre-optic view, laryngeal visibility was adequate in 64 (88.89%) patients in the neutral position and in 65 (90.28%) patients in the sniffing position with no statistically significant difference (Table 2).

**Table 2:** Fibre-optic view of the glottis with Cormack–Lehane classification (*n* = 72).

	Neutral position		Sniffing position		*p*-value
	*n*	%	*n*	%	
Grade					
1	42	58.33%	43	59.72%	
2	22	30.56%	22	30.56%	
3	7	9.72%	5	6.94%	
4	1	1.39%	2	2.78%	
Total	72	100.00%	72	100.00%	0.8783
Laryngeal view					
Adequate	64	88.89%	65	90.28%	
No adequate	8	11.11%	7	9.72%	
Total	72	100.00%	72	100.00%	0.785

In the fibre-optic view of the glottis assessed using the Brimacombe classification, laryngeal visibility was adequate in 68 (93%) patients in the neutral position and in 69 (95%) patients in the sniffing position, with no statistically significant difference (Table 3).

**Table 3:** Fibre-optic view of the glottis with Brimacombe classification (*n* = 72).

	Neutral position		Sniffing position		*p*-value
	*n*	%	*n*	%	
Score					
4	40	55.56%	39	54.17%	
3	18	25.00%	18	25.00%	
2	10	13.89%	12	16.67%	
1	4	5.56%	3	4.17%	
Total	72	100.00%	72	100.00%	0.817
Laryngeal view					
Adequate	68	94.44%	69	95.83%	
No adequate	4	5.56%	3	4.17%	
Total	72	100.00%	72	100.00%	0.6984

There was no statistically significant difference in the rate of success between the sniffing position (70 patients, 97.22% success rate) and the neutral position (67 patients, 93.06% success rate) during the first insertion attempt. In cases where the first attempt was unsuccessful, success was achieved in the second attempt for all the patients. Two patients required a second attempt in the sniffing position, while five patients required a second attempt in the neutral position (Table 4).

**Table 4:** Success, airway manoeuvres, and complication.

	Neutral position		Sniffing position	
Successful placement	72	100%	72	100%
Number of attempts				
1	67	93.06%	70	97.22%
2	5	6.94%	2	2.78%
Airway manoeuvres				
No one	43	59.72%	41	56.94%
ELM*	1	1.39%	1	1.39%
Jaw thrust	27	37.50%	29	40.28%
Relocation	1	1.39%	1	1.39%
Improved	23	82.14%	24	80.00%
Not improved	5	17.86%	6	20.00%
Complications				
No one	56		58	
Lips trauma	1		1	
Blood of SAD at time of removal	15		13	
Dental trauma	0		0	
Total (*n*)	16		14	
Incidence (%)	22.22%		19.44%	

*ELM: External laryngeal manipulation.

More than 50% of patients did not require any airway interventions to improve device placement. Jaw thrust was the most used airway manoeuvre and was used in 20% of the cases for the sniffing position and 17% of the cases for the neutral position; this manoeuvre did not improve vision in all the cases (Table 4).

Upper airway trauma, as evaluated by the presence of blood staining of the devices after their removal, was the most frequent complication (Table 5). The presence of blood was noted in 13 patients in the sniffing position and 15 patients in the neutral position following LMA removal. There was no statistically significant difference when comparing the presence of complications. There was no correlation by comparing Mallampati score, tryromental distance, intercisor gap, neck circumference, and fibre-optic scoring. All patients maintained an oxygen saturation ≥ 99%. Gastric tube insertion was successful in all the patients. Gastric distension did not occur in any patient.

**Table 5:** Variation in heart rate and blood pressure before and after colocation of vision mask.

Type of adverse events	*n*	%
Variation of mean blood pressure (mmHg)		
≥ 20%	15	20.83%
< 20%	57	79.17%
Variation of heart rate (bpm)		
≥ 20%	8	11.11%
< 20%	64	88.89%

Type of adverse events encountered.

Vision Mask insertion-related variations in the heart rate and blood pressure are shown in Table 5: 15 patients (20.83%) developed variation of mean blood pressure of more than ≥20% and variation of heart rate of more than ≥20% in eight patients (11.11%).

## 4 Discussion

This is the first study to the best of our knowledge evaluating a third-generation SAD.

The primary objective of this study was to compare the best glottis view in two head and neck positions with Vision Mask, a laryngeal mask video system. For this purpose, a standard pillow was not used to reach the sniffing position, and the position was reproduced by placing blankets under the head to horizontally align between the external auditory meatus and the sternal recess. The sniffing position has been recommended for conventional LMA insertion, but the height of the head in the sniffing position depends on the patient’s anatomy.

It is frequently observed that the sniffing position is utilised with varying head and neck angles in numerous studies, yet the alignment of the axes, namely, the oral–pharyngeal–laryngeal axes, are often overlooked.

In this study, it was not found that the sniffing position allows a better view of the glottic area using the Vision Mask device. The findings of the pilot study, which indicated that the sniffing position was associated with 100% good glottic view, were not corroborated. No difference was observed between the two positions.

The direct view of the larynx during the fitting of the device means that the position of the head does not influence the correct positioning of the mask.

A group of patients did not achieve adequate positioning of the device, but the device still allowed them to be well ventilated, confirming the findings of a previous report that there is no correlation between adequacy of ventilation and a low fibre-optic score (27). When evaluated using the CL classification, only the epiglottis was seen 9.72% of the patients and the laryngeal structures were not seen in 1.39% of the patients in the neutral position. In the sniffing position, these figures were 6.94 and 2.78%, respectively. Using the Brimacombe classification, the vocal cords were not visible in 5.56% of the patients (four) in the neutral position and 4.17% of the patients (three) in the sniffing position. The results were better than those obtained when the device placement was blind (9.4%) (25, 26).

Prior to the advent of third-generation SADs, the glottic area was evaluated by direct and indirect methods (12–17), and this device now gives the possibility of having a direct view of the glottic area.

In this study, we evaluated the glottic area in real time throughout the entirety of the procedure (60%) or the entirety of the anaesthesia (40%) with a video laryngeal mask system. Vision Mask allows a continuous side view while connected to the monitor.

Other studies have shown that different head positions during laryngeal mask insertion does not seem to impact the fibre-optic score, ventilation parameters, and the success rate of LMA placement (28, 29). Lim et al. (30) did not observe differences in glottis views between the supine and ramped positions. In this research, glottic view assessment was based on CL laryngeal view classification.

The use of different head heights with pillows has been employed in order to facilitate the placement of a laryngeal mask: ≤6 cm (30), 7 cm (28, 31), 8 cm (16, 29), or 14 cm (8). It was found that the extremes—3 cm (32) and 14 cm (8)—allowed successful insertion of the LMA.

A meta-analysis shows that the flexed neck position significantly improves airway sealing but adversely affects ventilation and the fibre-optic view with most SADs. Although neck extension significantly reduced airway sealing, it did not affect ventilation or the fibre-optic view (9).

It has also been found that a difficult airway and head position do not affect the ease of insertion of a LMA ProSeal (PLMA) and fibre-optic score (16).

In the study, LMA Vision Mask were successfully positioned in the neutral position 93% of the time and in the sniffing position 97% of the time on the first attempt. The success rate for insertion at the first attempt in the LMA Classic has been reported to be between 77 and 97% (17) depending on the experience of the anaesthesiologist and suboptimal positioning of the LMA occurs in 30–66% of the cases (32) and in 50–80% of the cases (14) according to different authors with the blind placement.

In this study, we used a fibre-optic scoring system for standardised evaluation of the LMA position following its insertion into the hypopharynx proposed by Brimacombe and Berry and compared the glottic view with that found with the CL classification, which is used for direct laryngoscopy. Most studies use the Brimacombe score (33–35) and very few the Cormack–Lehane laryngeal view classification (30). When the two classifications were compared, the glottic view was found to be consistent in 88–89% of cases using the CL classification and in 93–95% of cases using the Brimacombe classification. Device placement was considered optimal when the fibre-optic score with Brimacombe was grade 2 to 4 or Cormack–Lehane 1 or 2. In both situations, the vocal cords are visible, either fully or partially.

The proper placement and functioning of the laryngeal mask has been evaluated through clinical tests and using different equipment that allowed visualisation of the glottic area to confirm that the procedure was well performed. By moving from a blind procedure to visualization of the anatomy, we assume that it increases safety in airway management, although this visualisation of the glottic area is not directly related to the sealing device. The efficacy of the seal or tightness may vary depending on the individual patient’s laryngopharyngeal anatomy, in addition to the anatomical placement of the LMA.

Campbell et al. (12) used fibre-optic examination to compare the traditional blind insertion technique of LMA placement with direct visual placement using a laryngoscope. They reported that the appropriate positioning of the LMA had been achieved in 91.5% of the patients in the direct visual placement group compared to 42% in the blind insertion group. However, other researchers indicate that laryngoscope-guided insertion is not superior to blind insertion in terms of achieving proper anatomical placement of the LMA, since the fibre-optic position scores were similar for both techniques (13). Chandal et al. (36) concluded that the blind insertion is easier and simpler method for insertion of LMA and has a reasonable success during insertion, and recommended its use.

The visualisation of the glottis during insertion of the Vision Mask allows immediate detection and correction of inadequate cuff inflation, incorrect device size, glottis distortion, or epiglottis within the cuff. Any problems can be resolved by adjusting the head position, changing the LMA, or using an additional manoeuvre. However, it is important to note that this method may not be successful in all cases.

On the other hand, the correct placement of an SAD is also dependent in the material of the device (37).

In our study, the frequency of a fibre-optic score of more than 2 (suboptimal anatomic position) was 11.11% in the neutral position and 9.72% in the sniffing position with CL classification and the frequency of a fibre-optic score less than 2 (suboptimal position) was 5.56% in the neutral position and 4.17% in the sniffing position with Brimacombe classification (using neuromuscular relaxation with 0.6 mg/kg of Rocuronio). In a study conducted by Brimacombe and Keller, it was found that poor vision was present in one out of 60 patients with LMA and in four out of 60 patients with ProSeal laryngeal mask airway (PLMA). The study used vecuronium at a dose of 0.1 mg/kg (32).

Other studies find a higher percentage of inadequate LMA position assessed with fibroscopy. In 148 patients who had successful PLMA insertion, Jun et al. (34) found 77 patients (52%) to have a suboptimal position (fibre-optic score <3) similar to the results of Brimacombe et al. (50.3%) (38). In these two studies, the neuromuscular relaxation dose was low (rocuronium 0.3 mg/kg) or the patients were not relaxed.

Jun et al. (16) found that changing the head position after ProSeal insertion did not significantly change the fibre-optic score. In this study, the frequency of vocal cord visibility was 87.2–93.9% in all groups, which was similar to the results of Brimacombe and Keller (33) (93.3%).

Cook et al. (39) reported 30 manoeuvres necessary to optimise the airway patency in 24 patients. In this study, it was necessary to perform airway manoeuvres 39–42% of the time to improve glottic view using Vision Mask in 72 patients.

Jaw thrust was the most frequently used manoeuvre, and with this manoeuvre, the epiglottis is raised and the distance between the posterior aspect of the tongue and posterior pharyngeal wall was increased. There were no significant differences in the number of manipulations between both positions.

LMA is associated with lower complication rates. Severe traumatic complications occur rarely (40). In this study, we found that the most frequent complication was the presence of blood in the Vision Mask, which was observed on its removal. Additionally, lip lesions were observed in one patient from each different position. Although the reported complications are not frequent and not very serious, a significantly higher blood staining on the mask has been noted with Vision Mask. The total adverse event rate was 22.22% in patients in the neutral position and 19.44% in patients in the sniffing position, and no severe complications were observed.

A systematic review and meta-analysis of randomised controlled trials, comparing the LMA Supreme with the LMA ProSeal (37), and a multicentre study comparing the ProSeal with the Classic LMA, revealed that the incidence of more frequent adverse event is blood staining on the LMA (38). Other complications were vomiting, sore throat, dysphagia, dysphonia, and laryngospasm (37).

## 5 Limitations

Our study has some limitations. All Vision Mask devices were applied by only one author, so the results express the author’s experience with the Vision Mask.

In this observational study, no randomisation of patients was performed because the same device was placed in the sniffing position and the neutral position for the same patient.

We could consider the evaluation of the glottis view, although blind, carried out by a single researcher as a limitation. It is not possible to make a comparison between the complications of the device being placed twice in the same patient. Our results may not be applicable to patients with spontaneous ventilations.

To summarise, a SAD with vision allows us a correct placement of the device in real-time and reposition if necessary.

In this study, we evaluated the glottic view using two different head–neck positions: the sniffing position and a neutral position with SAD Vision Mask. We found that an adequate sniffing position did not result in a better glottic view than a neutral position. The additional manoeuvres for a better glottic view were similar in both positions. In conclusion, the head–neck position does not influence the placement of a third-generation SAD.

## Data Availability

The raw data supporting the conclusions of this article will be made available by the authors, without undue reservation.
